# Public attitudes towards COVID-19 vaccine mandates and vaccine certificates in Canada: a time series study

**DOI:** 10.1186/s13690-024-01259-8

**Published:** 2024-03-11

**Authors:** David T. Zhu, Steven Hawken, Mohamed Serhan, Frank Graves, Jeff Smith, Kumanan Wilson

**Affiliations:** 1 Clinical Epidemiology Program, Ottawa Hospital Research Institute, Ottawa, ON Canada; 2 Virginia Commonwealth School of Medicine, Richmond, VA USA; 3 School of Epidemiology and Public Health, University of Ottawa, Ottawa, ON Canada; 4 EKOS Research Associates Inc., Ottawa, ON Canada; 5 Department of Pediatrics, University of Ottawa, Ottawa, ON Canada; 6 Department of Medicine, University of Ottawa, Ottawa, ON Canada; 7 Bruyère Research Institute, Ottawa, ON Canada; 8 O’Neill Institute for National and Global Health Law, Georgetown University, Washington, D.C USA; 9 Ottawa Hospital, Civic Campus, Administrative Services Building, 1053 Carling Avenue, Box 684, Ottawa, Ontario K1Y 4E9 Canada

**Keywords:** COVID-19, Vaccine, Vaccine mandate, Vaccination certificate, Attitude

## Abstract

**Introduction:**

Since the beginning of the pandemic, numerous public health measures such as COVID-19 vaccines, vaccine mandates and vaccination certificates have been introduced to mitigate the spread of COVID-19. Public opinion and attitudes towards these measures have fluctuated in response to the dynamic political, social, and cultural landscape of the pandemic.

**Methods:**

We conducted a time-series study consisting of national cross-sectional surveys between November 2021 to March 2022 to evaluate the Canadian public’s attitudes towards COVID-19 vaccine mandates and vaccine certificates.

**Results:**

When examining public sentiment towards COVID-19 vaccine certificates and proof of vaccination measures, there was a shift in responses over time. The proportion of participants “strongly supporting” these measures decreased from 66.0 to 43.1% between W25(Capacity Limits), −W32 (Mask Mandate Removed), whereas “strongly oppose” was the second most common response and rose from 15.9 to 20.6% during this same time period. Concurrently, when examining participants views surrounding mandates, many participants believed that their province was reopening at “about the right pace”, which remained relatively stable over time (33.0–35.4%) between W28 (Emergency Act)–W32 (Mask Mandate Removed).

**Conclusion:**

Our study’s findings on the public’s attitudes towards COVID-19 vaccine mandates and vaccine certificates in Canada may aid to guide and streamline the implementation of future similar public health interventions. Future research should include extended follow-up and a more comprehensive examination of trust in government institutions and polarized perspectives on vaccine mandates.

**Supplementary Information:**

The online version contains supplementary material available at 10.1186/s13690-024-01259-8.

**Table:** 

Text box 1. Contributions to the literature	
• Our study provides insights into public perception surrounding COVID-19 vaccine mandates/certificates in Canada over time	
• We examined the impact of social and cultural events on public perceptions and attitudes	
• Understanding the challenges and public perception surrounding vaccine mandates/certificated can aid policy makers in implementing future measures	

## Introduction

Myriad public health measures have been implemented by governments around the world to curb the spread of COVID-19. These include, but are not limited to, personal protective equipment (PPE), COVID-19 vaccines, vaccine certificates, stay-at-home orders and quarantine measures, restrictions on non-essential businesses and social gatherings, and more [1, 2]. Vaccine certificates, specifically, have generally been successful at increasing COVID-19 vaccine uptake among the general population [3, 4], although their relative degree of effectiveness in particular regions varies according to social and environmental factors such as vaccine hesitancy, the local COVID-19 burden, and the timing and implementation strategy [3, 5]. For instance, one modeling study found that vaccine mandates increased COVID vaccination rates in six provinces, ranging from approximately 4.4 percentage-points in British Columbia to 8.2 percentage-points in Alberta [6].

However, the introduction of COVID-19 vaccine mandates has been criticized for restricting individual autonomy and freedom of choice to vaccinate, representing a key ethical issue [7, 8]. Critics argue that vaccine mandates are coercive and create unjust punishments for individuals who are ambivalent, hesitant, or hostile to receiving COVID-19 vaccines, such as travel and work restrictions [9]. Public support and attitudes towards COVID-19 vaccine mandates have evolved over time according to factors such as macroeconomic changes, significant social and cultural events, political influences, and more [10, 11].

To the best of our knowledge, there are no previous studies that have examined the evolution of public attitudes towards COVID-19 vaccine mandates over time in the Canadian context. Our time-series study (descriptive) addresses this gap by exploring geographic and temporal trends in Canadians’ attitudes towards vaccine mandates and public health measures during the COVID-19 pandemic. These findings may have important policy implications for the implementation of vaccine mandates during future pandemics.

## Methods

### Survey characteristics and delivery

A series of national cross-sectional surveys were conducted between November 2021 to March 2022 to evaluate Canadian public attitudes towards COVID-19 vaccine mandates. Within this timeframe, there were eight survey waves, defined as the following: W25 (November 16–25, 2021) (Capacity Limits), W26 (December 15–21, 2021,Border Measures), W27 (January 18–24, 2022, Freedom Convoy Began) W28 (February 2–9, 2022, Emergency Act) W29 (February 16–21, 2022, Freedom Convoy Ended), W30 (February 25–March 3, 2022, Proof of Vaccination Ended), W31 (March 9–13, 2022, Statement to Remove All COVID Restrictions), and W32 (March 17–22, 2022, Mask Mandate Removed). The study sample was closed meaning that the same pool of participants was used to draw the sample for each survey. No participants entered or exited the sample.

The survey was conducted by a national polling company (EKOS) using a probability panel and random sampling methods to ensure the final sample was representative of the target general adult population in Canada. The consistency of the data was ensured by cross referencing the data we collected to external official statics validating the accuracy and reliability of our sample. We employed both a scale and questionnaire to assess individuals’ attitudes in our survey. Each question was thoroughly pretested to ensure that the appropriate scale was used to gather the appropriate data at hand. We used Random Iterative Method (RIM) weighting to adjust for any departures in sociodemographic characteristics from census parameters to ensure the representativeness of the sample [12]. Furthermore, a live operator verified the identity of each participant enrolled in the sample and removed any duplicate or fraudulent responses.

Eligible individuals were those living in Canada, 18 years or older, and English- or French-speaking. Informed consent was collected from each participant at the time of recruitment into the probability panel, prior to participating in the online survey. For each of the eight waves, the survey was validated using a convenience sample of participants (*n* = 10) from the panel by members of the research team to ensure that the survey questions were clearly understood. Henceforth, the survey was electronically sent to participants through email, with frequent prompts if the participant did not yet respond. The response rates were calculated by dividing the total number of survey completions by the total number of email invitations sent to valid emails addresses of eligible participants.

The survey questions were related to social and demographic information, vaccination history (including whether or not they had received the first, second, and third doses of the COVID-19 vaccines), perceptions towards the safety and efficacy of COVID-19 vaccines, and attitudes towards the vaccine mandates/certificates and public health measures implemented in Canada to curb the spread of COVID-19. Notably, the W28 (Emergency Act) survey also included questions about the Freedom Convoy, a protest movement that began in November 2021 in Ottawa, Canada, wherein thousands of truckers and other protestors expressed their opposition to COVID-19 vaccine mandates and other pandemic-related restrictions. This was a significant societal event during the COVID-19 pandemic that may have influenced public attitudes towards vaccination. The complete list of survey questions is provided in (Table S1).

This study received ethical approval by the Bruyère Research Institute Research Ethics Board (REB# M16–22-007) and the Ottawa Hospital Research Ethics Board (REB# 20220115-01H).

### Statistical analysis

We calculated descriptive statistics for participant sociodemographic characteristics as well as survey responses across the eight waves. Reponses related to the Freedom Convoy protests were only reported for the W28 (Emergency Act) survey wave. We fitted multivariable logistic regression models to for each survey wave to evaluate the association between sociodemographic predictors and survey responses for perceptions of proof of COVID-19 vaccination measures in Canada. The outcome variable was dichotomized to those who “support” or “oppose” proof of COVID-19 vaccination measures. Statistical analyses were performed in R (V4.2.1) and IBM SPSS (V2022). Graphical visualizations were produced with Microsoft Excel (V16.72).

## Results

### Participant characteristics

Participant sociodemographic information for each survey are shown in (Table 1). The sample predominantly consisted of individuals aged 25 years or older (97.0–98.2%), were living in urban areas (39.2–40.5%), were from Ontario (34.4–38.7%), did not identify as a minority (63.8–68.3%), and had completed some sort of postsecondary education such as a bachelor’s degree (24.4–27.9%) or college/non-university degree (21.3–23.0%). Approximately half of our sample had annual household incomes of $60,000–$219,999. There was a relatively even balance of males (45.8–50.1%) and females (47.1–52.3%) in our sample. The response rate ranged from 10.2–12.6% across the survey waves. See (Table 1) for the response rate of each individual survey.

**Table 1:** General sample characteristics

Survey Responses	W25 *n* (%)	W26 *n* (%)	W27 *n* (%)	W28 *n* (%)	W29 *n* (%)	W30 *n* (%)	W31 *n* (%)	W32 *n* (%)
**Total respondents (N)**	1251 (100)	1015 (100)	1225 (100)	1004 (100)	1003 (100)	1097 (100)	1035 (100)	1048 (100)
**Response rate (%)**	11.1	11.0	11.7	12.6	12.5	10.7	11.0	10.2
**Age (years)**								
18–24	26 (2.1)	19 (1.9)	36 (2.9)	23 (2.3)	30 (3.0)	28 (2.6)	20 (1.9)	19 (1.8)
25–34	282 (22.5)	232 (22.9)	275 (22.4)	224 (22.3)	236 (23.5)	205 (18.7)	247 (23.9)	218 (20.8)
35–44	207 (16.5)	151 (14.9)	193 (15.8)	160 (15.9)	181 (18.0)	195 (17.8)	159 (15.4)	213 (20.3)
45–54	214 (17.1)	167 (16.5)	199 (16.2)	176 (17.5)	155 (15.5)	194 (17.7)	166 (16.0)	189 (18.0)
55–64	200 (16.0)	178 (17.5)	222 (18.1)	177 (17.6)	157 (15.7)	192 (17.5)	181 (17.5)	160 (15.3)
65 or older	280 (22.4)	213 (21.0)	251 (20.5)	204 (20.3)	201 (20.0)	231 (21.1)	228 (22.0)	219 (20.9)
Don’t know/no response	42 (3.4)	55 (5.4)	49 (4.0)	40 (4.0)	43 (4.3)	52 (4.7)	34 (3.3)	30 (2.9)
**Gender**								
Male	618 (49.4)	509 (50.1)	614 (50.1)	486 (48.4)	491 (49.0)	518 (47.2)	518 (50.0)	480 (45.8)
Female	615 (49.2)	478 (47.1)	586 (47.8)	490 (48.8)	485 (48.4)	550 (50.1)	496 (47.9)	548 (52.3)
Other	8 (0.6)	15 (1.5)	16 (1.3)	14 (1.4)	12 (1.2)	13 (1.2)	14 (1.4)	13 (1.2)
Don’t know/no response	9 (0.7)	13 (1.3)	9 (0.7)	14 (1.4)	15 (1.5)	16 (1.5)	7 (0.7)	7 (0.7)
**Rurality**								
Rural	299 (23.9)	214 (21.1)	275 (22.4)	217 (21.6)	228 (22.7)	247 (22.5)	234 (22.6)	222 (21.2)
Suburban	447 (35.7)	383 (37.7)	449 (36.7)	365 (36.4)	376 (37.5)	416 (37.9)	374 (36.1)	404 (38.5)
Urban	491 (39.2)	404 (39.8)	473 (38.6)	407 (40.5)	386 (38.5)	413 (37.6)	411 (39.7)	414 (39.5)
Don’t know/no response	14 (1.1)	14 (1.4)	28 (2.3)	15 (1.5)	13 (1.3)	21 (1.9)	16 (1.5)	8 (0.8)
**Province**								
British Columbia	167 (13.3)	136 (13.4)	173 (14.1)	117 (11.7)	133 (13.3)	159 (14.5)	156 (15.1)	145 (13.8)
Alberta	148 (11.8)	127 (12.5)	116 (9.5)	130 (12.9)	122 (12.2)	134 (12.2)	116 (11.2)	144 (13.7)
Saskatchewan	26 (2.1)	33 (3.3)	31 (2.5)	35 (3.5)	33 (3.3)	30 (2.7)	34 (3.3)	40 (3.8)
Manitoba	41 (3.3)	25 (2.5)	39 (3.2)	33 (3.3)	26 (2.6)	29 (2.6)	43 (4.2)	39 (3.7)
Ontario	478 (38.2)	370 (36.5)	474 (38.7)	376 (37.5)	396 (39.5)	394 (35.9)	378 (36.5)	360 (34.4)
Quebec	283 (22.6)	219 (21.6)	281 (22.9)	205 (20.4)	199 (19.8)	252 (23.0)	182 (17.6)	230 (21.9)
New Brunswick	31 (2.5)	31 (3.1)	24 (2.0)	24 (2.4)	26 (2.6)	24 (2.2)	32 (3.1)	18 (1.7)
Nova Scotia	50 (4.0)	38 (3.7)	44 (3.6)	29 (2.9)	38 (3.8)	37 (3.4)	48 (4.6)	37 (3.5)
Prince Edward Island	5 (0.4)	3 (0.3)	5 (0.4)	3 (0.3)	2 (0.2)	4 (0.4)	3 (0.3)	4 (0.4)
Newfoundland and Labrador	18 (1.4)	9 (0.9)	15 (1.2)	14 (1.4)	9 (0.9)	10 (0.9)	17 (1.6)	13 (1.2)
Yukon	2 (0.2)	14 (1.4)	11 (0.9)	18 (1.8)	8 (0.8)	16 (1.5)	10 (1.0)	13 (1.2)
Northwest Territories	1 (0.1)	8 (0.8)	6 (0.5)	14 (1.4)	4 (0.4)	4 (0.4)	10 (1.0)	3 (0.3)
Nunavut	0 (0.0)	2 (0.2)	3 (0.2)	3 (0.3)	4 (0.4)	2 (0.2)	3 (0.3)	2 (0.2)
Don’t know/no response	1 (0.1)	0 (0.0)	3 (0.3)	3 (0.3)	3 (0.3)	2 (0.2)	3 (0.3)	0 (0.0)
**Annual household income**								
< $10,000	17 (1.4)	12 (1.2)	8 (0.7)	10 (1.0)	15 (1.5)	6 (0.5)	9 (0.9)	8 (0.8)
$10,000–$19,999	25 (2.0)	24 (2.4)	31 (2.5)	22 (2.2)	26 (2.6)	34 (3.1)	21 (2.0)	13 (1.2)
$20,000–$29,999	45 (3.6)	31 (3.1)	63 (5.1)	38 (3.8)	42 (4.2)	35 (3.2)	37 (3.6)	36 (3.4)
$30,000–$39,999	55 (4.4)	45 (4.4)	70 (5.7)	43 (4.3)	61 (6.1)	45 (4.1)	50 (4.8)	53 (5.1)
$40,000–$49,999	67 (5.4)	53 (5.2)	69 (5.6)	47 (4.7)	51 (5.1)	54 (4.9)	55 (5.3)	49 (4.7)
$50,000–$59,999	86 (6.9)	69 (6.8)	76 (6.2)	49 (4.9)	65 (6.5)	70 (6.4)	71 (6.9)	63 (6.0)
$60,000–$79,999	145 (11.6)	114 (11.2)	145 (11.8)	120 (12.0)	108 (10.8)	126 (11.5)	116 (11.2)	122 (11.6)
$80,000–$99,999	138 (11.0)	116 (11.4)	133 (10.9)	106 (10.6)	111 (11.1)	124 (11.3)	105 (10.1)	107 (10.2)
$100,000–$119,999	160 (12.8)	115 (11.3)	137 (11.2)	118 (11.8)	118 (11.8)	131 (11.9)	106 (10.2)	126 (12.0)
$120,000–$159,999	147 (11.8)	133 (13.1)	135 (11.0)	156 (15.5)	127 (12.7)	133 (12.1)	146 (14.1)	159 (15.2)
$160,000–$219,999	125 (10.0)	82 (8.1)	113 (9.2)	90 (9.0)	96 (9.6)	113 (10.3)	101 (9.8)	99 (9.4)
$220,000 or more	70 (5.6)	79 (7.8)	94 (7.7)	69 (6.9)	52 (5.2)	77 (7.0)	78 (7.5)	78 (7.4)
Don’t know/no response	171 (13.7)	142 (14.0)	151 (12.3)	136 (13.5)	131 (13.1)	149 (13.6)	140 (13.5)	135 (12.9)
**Minority status**								
Visible minority	116 (9.3)	118 (11.6)	167 (13.6)	135 (13.4)	128 (12.8)	118 (10.8)	127 (12.3)	118 (11.3)
Indigenous	34 (2.7)	29 (2.9)	32 (2.6)	16 (1.6)	27 (2.7)	32 (2.9)	29 (2.8)	30 (2.9)
Disability	104 (8.3)	90 (8.9)	107 (8.7)	78 (7.8)	94 (9.4)	90 (8.2)	101 (9.8)	98 (9.4)
2SLGBTQ+	84 (6.7)	70 (6.9)	72 (5.9)	50 (5.0)	50 (5.0)	58 (5.3)	53 (5.1)	53 (5.1)
None of the above	855 (68.3)	655 (64.5)	782 (63.8)	677 (67.4)	642 (64.0)	739 (67.4)	666 (64.3)	697 (66.5)
Don ‘t know/no response	58 (4.6)	53 (5.2)	65 (5.3)	48 (4.8)	62 (6.2)	60 (5.5)	59 (5.7)	52 (5.0)
**Highest educational level**								
Grade 8 or less	2 (0.2)	1 (0.1)	2 (0.2)	0 (0.0)	4 (0.4)	3 (0.3)	2 (0.2)	1 (0.1)
Some high school	19 (1.5)	22 (2.2)	23 (1.9)	16 (1.6)	23 (2.3)	14 (1.3)	20 (1.9)	19 (1.8)
High school diploma	214 (17.1)	176 (17.3)	209 (17.1)	168 (16.7)	158 (15.8)	172 (15.7)	173 (16.7)	149 (14.2)
Apprenticeship/trade diploma	72 (5.8)	71 (7.0)	86 (7.0)	66 (6.6)	70(7.0)	63 (5.7)	62 (6.0)	63 (6.0)
College, CEGEP, or other non-university diploma	288 (23.0)	220 (21.7)	277 (22.6)	238 (23.7)	216 (21.5)	251 (22.9)	238 (23.0)	223 (21.3)
University diploma (non-Bachelor’s)	82 (6.6)	76 (7.5)	92 (7.5)	76 (7.6)	79 (7.9)	93 (8.5)	76 (7.3)	69 (6.6)
Bachelor’s degree	316 (25.3)	248 (24.4)	318 (26.0)	252 (25.1)	256 (25.5)	270 (24.6)	282 (27.2)	292 (27.9)
Graduate degree	241 (19.3)	187 (18.4)	201 (16.4)	168 (16.7)	181 (18.0)	213 (19.4)	168 (16.2)	220 (21.0)
Don’t know/no response	17 (1.4)	14 (1.4)	17 (1.4)	20 (2.0)	16 (1.6)	18 (1.6)	14 (1.4)	12 (1.1)

### COVID-19 vaccination trends over time

**Fig. 1:**
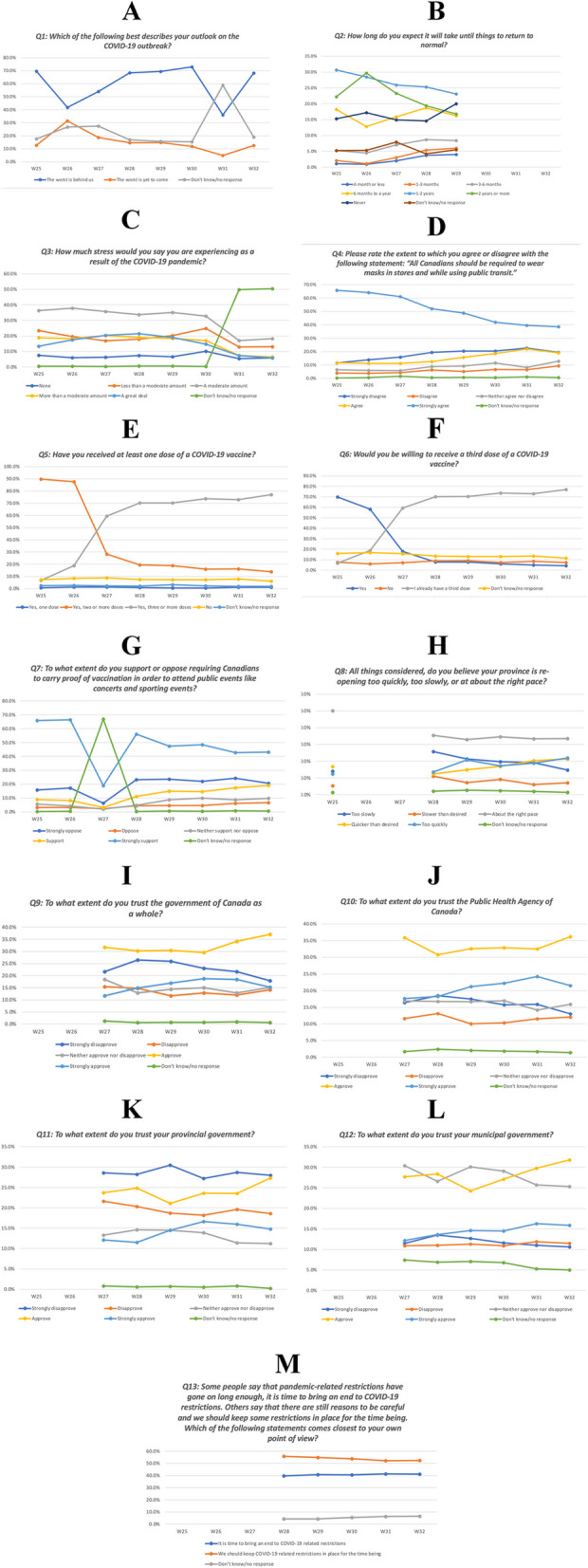
Time trends for recurring survey questions

We observed a relationship where over time as the number of individuals who got vaccinated increased, individuals who were willing to get vaccinated decreased. This suggest that individuals who were wanted a vaccine were able to acquire one. From W25(Capacity Limits) to W32 (Mask Mandate Removed), willingness to receive a third dose of the COVID-19 vaccine decreased by 65.6 percentage-points, which is similar in magnitude to the 70.3 percentage-point increase in prevalence of those who received a third dose. This shift was largest between W25 (Capacity Limits), −W27 (Fig. 1E, F) (Table S1). The prevalence of those who were unwilling to receive a third dose of the COVID-19 vaccine did not vary considerably over time, ranging from 6.1–9.9% (Fig. 1E, F) (Table S1).

Over time people were willing to vaccinate decreased, and people who were vaccinated increased suggesting that people who wanted to receive a vaccine were able to get one.

### Public attitudes towards the COVID-19 pandemic over time

Participants’ outlook on the COVID-19 pandemic generally improved over time. From W26 (Border Measures) to W32 (Mask Mandate Removed), the prevalence of those who perceived that “the worst is behind us” increased by 26.5 percentage-points. In contrast, the prevalence of those who perceived that “the worst is yet to come” decreased by 18.9 percentage-points (Fig. 1A) (Table S1). Similarly, from W25 (Capacity Limits), to W29 (Freedom Convoy Ended), the prevalence of those who expected that society will “return to normal” in the short-term (within 6 months or less) increased slightly by 2.9–3.9 percentage-points, whereas the prevalence of those who expected that society will “return to normal” in the long-term (within 1 year or more) decreased 5.4–7.6 percentage-points (Fig. 1B) (Table S1).

Participants’ self-reported stress levels were relatively similar to one another and remained fairly stable over time, such that participants most commonly reported a persistent “moderate” level of stress experienced during the COVID-19 pandemic (Fig. 1C) (Table S1). There appeared to be a convergence of self-reported stress levels over the study period.

### Public attitudes towards COVID-19 vaccine mandates over time

With regards to public attitudes towards wearing masks in stores and while using public transit, participants most commonly reported that they “strongly agree” with these measures, although this decreased from 65.7 to 38.6% between W25 (Capacity Limits), and W32 (Mask Mandate Removed) (Fig. 1D) (Table S1). In contrast, those who “strongly disagree” with these measures was the second most common response, which increased from 11.6 to 19.4% during this same time period (Fig. 1D) (Table S1). Similarly, with regards to public attitudes towards COVID-19 vaccine certificates and proof of vaccination measures, “strongly support” was the most common response and fell from 66.0 to 43.1% between W25(Capacity Limits), −W32 (Mask Mandate Removed), whereas “strongly oppose” was the second most common response and rose from 15.9 to 20.6% during this same time period (Fig. 1G) (Table S1).

The majority of participants believed that their province was reopening at “about the right pace”, which remained relatively stable over time (33.0–35.4%) between W28 (Emergency Act)–W32 (Mask Mandate Removed) (Fig. 1H) (Table S1). Amongst participants who did not believe that their province was reopening at the right pace, they increasingly believed that the reopening was at a faster pace than desired. From W28 (Emergency Act) to W32 (Mask Mandate Removed), the prevalence of participants who believed that their province was reopening “too slowly” decreased by 10.9%, whereas the prevalence of those who believed that the reopening “quicker than desired” or “too quickly” increased by 8.8 and 8.4%, respectively (Fig. 1H) (Table S1). Notably, the intersection between those that responded “too slowly” vs. those that responded “quicker than desired” or “too quickly” occurred around W31 (Statement to Remove All COVID Restrictions) at a prevalence of approximately 19% (Fig. 1H) (Table S1). A slight majority of participants believed that “we should keep COVID-19 related restrictions in place for the time being”, which remained fairly constant over time, ranging between 52.3–55.8% (Fig. 1M) (Table S1). In contrast, 39.8–41.4% of participants believed that “it is time to bring an end to COVID-19 related restrictions”, which also remained fairly constant over time (Fig. 1M) (Table S1).

### Trust and approval towards government agencies over time

Over time, participants trust in the various levels of government and the Public Health Agency of Canada varied. In general, trust and approval towards the three different levels of government and PHAC improved.

With respect to PHAC, participants “approve” response rate increased 5.4% over time (from W28 (Emergency Act) to W32 (Mask Mandate Removed)) and the “strongly approve” response rate increased by 3.2% during the same time period (Fig. 1J) (Table S1). The “disapproval” response rate remained steady. However, the percentage of participants who stated they “strongly disapprove” decreased 5.5% over time (W28 (Emergency Act) to W32 (Mask Mandate Removed)) (Fig. 1J) (Table S1).

With respect to the federal government, the percentage of participants who responded that they “approve” increased 6.9% from W28 (Emergency Act) to W32 (Mask Mandate Removed) and the percentage who responded that they “strongly disapprove” decreased by 8.6% during the same time period (Fig. 1I) (Table S1).

With respect to the municipal government, the percentage of participants who stated that they “approve” initially decreased by 3.4% from W27 (Freedom Convoy Began) to W29 (Freedom Convoy Ended) but increased by 7.5% from W29 (Freedom Convoy Ended) to W32 (Mask Mandate Removed) (Fig. 1L) (Table S1). The percentage of participants who stated that they “neither approve nor disapprove” decreased by 5.1% from W29 (Freedom Convoy Ended) to W32 (Mask Mandate Removed) (Fig. 1L) (Table S1). Disapproval remained steady, but the percentage of participants who stated that they “strongly disapprove” decreased slightly by 2.9% from W28 (Emergency Act) to W32 (Mask Mandate Removed). The percentage of participants who stated that they “strongly approved” rose steadily by 3.7% between W27 (Freedom Convoy Began) and W32 (Mask Mandate Removed) (Fig. 1L) (Table S1).

With respect to the provincial government, the percentage of participants who stated that they “strongly disapprove,” was the most common response and this remained constant with a slight decrease of 0.6% from W27 (Freedom Convoy Began) to W32 (Mask Mandate Removed) (Fig. 1K) (Table S1). The percentage of participants who stated that they “approve” among participants initially dropped by 2.6% from W27 (Freedom Convoy Began) to W29 (Freedom Convoy Ended) but then increased by 6.2% from W29 (Freedom Convoy Ended) to W32 (Mask Mandate Removed). The percentage of participants who stated that they “strongly approve” increased by 2.7%, and those who stated that they “disapprove” decreased by 3.0% during this period (Fig. 1K) (Table S1).

### Public attitudes towards freedom convoy protestors

**Fig. 2:**
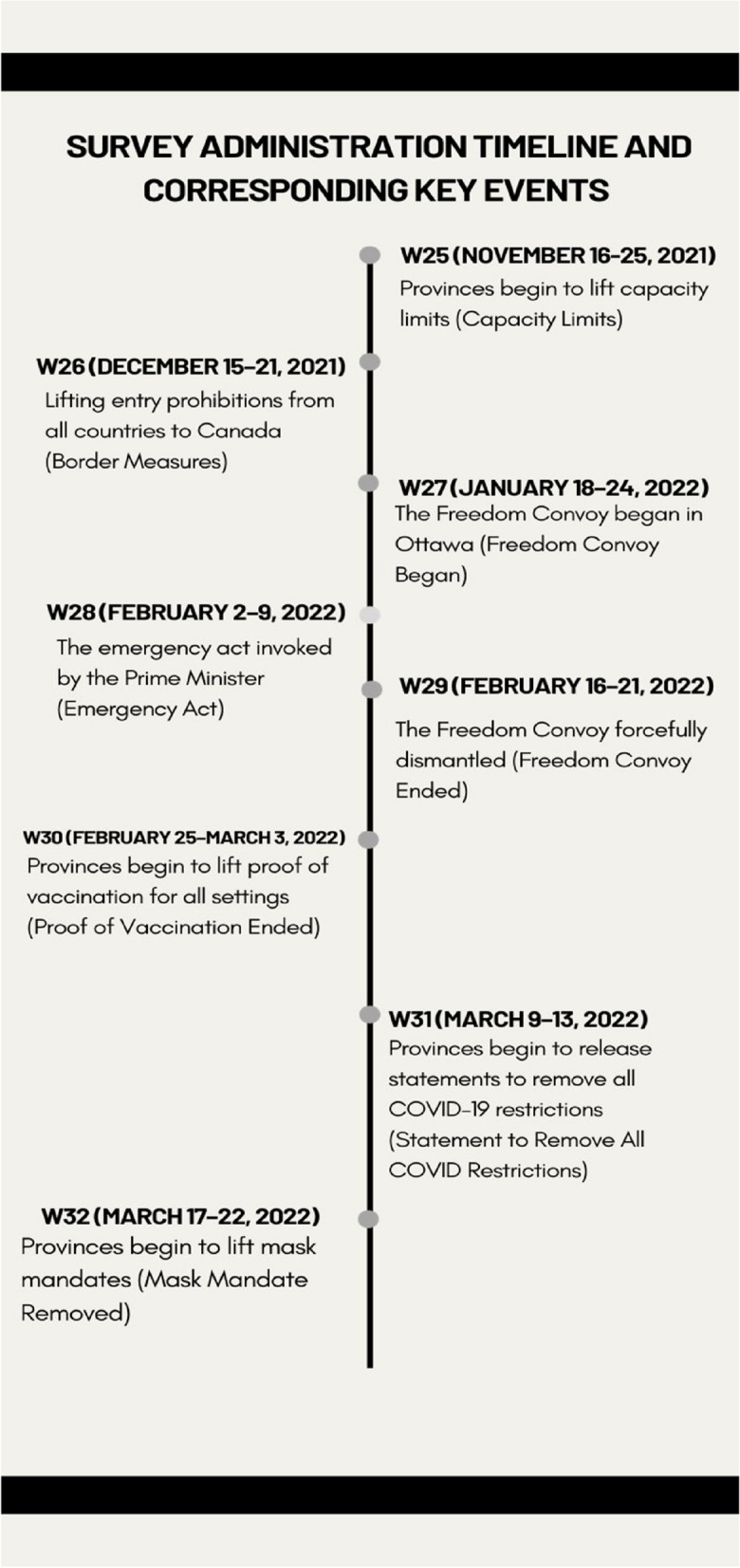
Timeline of survey administration and coinciding key social events

Participants’ support for the Freedom Convoy protests was highly polarized. The vast majority of respondents were aware of this protest, with 48.3 and 44.1% following this protest “very closely” and “somewhat closely”, respectively (Fig. 2B) (Table S2). The majority of respondents “strongly oppose” (51.9%) the Freedom Convoy protests, whereas 20.3% “strongly support” the protests (Fig. 2C) (Table S2). Similarly, the majority of respondents “strongly disagree” (58.7%) with supporting a similar Freedom Convoy protest in their own community, whereas 17.5% “strongly agree” with this proposition (Fig. 2D) (Table S2). Further, 24.5% of respondents responded that they “strongly support” mandating proof of vaccination for truckers entering Canada from the United States, whereas 14.1% responded that they “strongly oppose” these measures. However, caution should be taken when interpreting these findings due to the substantial proportion of missing data (50.1%) (Fig. 2A) (Table S2). The majority of participants responded that they “strongly oppose” (60.8%) the Freedom Convoy organizers’ “memorandum of understanding” that called upon the Governor General to override vaccine mandates, whereas 14.2% responded that they “strongly support” this memorandum (Fig. 2G) (Table S2).

**Fig. 3:**
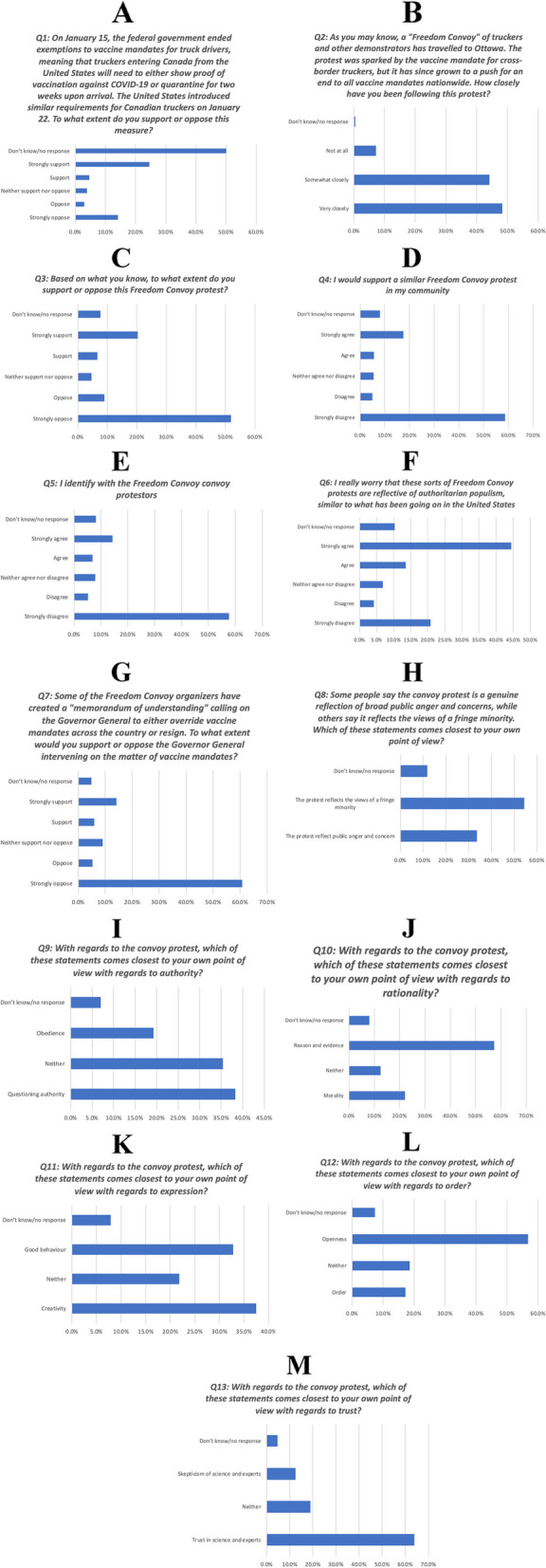
Bar plots for Freedom Convoy questions

We also observed polarization in participants’ identity congruence with the Freedom Convoy protestors. The majority of respondents responded that they “strongly disagree” (57.7%) with having a sense of shared identity with the Freedom Convoy protestors, whereas 14.3% responded that they “strongly agree” with this belief (Fig. 2E) (Table S2). With regards to authority, participants reported that the Freedom Convoy protests reflected their own views on “questioning authority” (38.3%) nearly two-fold more than “obedience” (19.3%) (Fig. 2I) (Table S2). Participants rated “reason and evidence” (57.4%) nearly three-fold more commonly than “morality” (22.1%) as the most important factor when evaluating the protests (Fig. 2J) (Table S2). Interestingly, with regards to self-expression, there was a similar prevalence of participants that reported that these protests reminded them of their own perspectives on “creativity” (37.5%) and on “good behavior” (32.8%) (Fig. 2K) (Table S2). With regards to sense of order, participants reported that these protests reflected their own perspectives on “order” (56.8%) more-than-three-fold more than on “openness” (17.2%) (Fig. 2L) (Table S2). Finally, participants rated “trust in science and experts” (63.9%) more-than-five-fold more than “skepticism of science and experts” (12.5%) as the most important factor when evaluating the protests (Fig. 2M) (Table S2).

Similarly, participants’ perceptions of the ideological principles of the Freedom Convoy protests was also highly polarized. Further, participants most commonly responded that they “strongly agree” (44.4%) with the belief that the Freedom Convoy protests reflect authoritarian populism in a similar manner to recent social and political events in the United States, whereas 20.8% of respondents responded that they “strongly disagree” with this belief (Fig. 2F) (Table S2). Similarly, the majority of participants believe that the protests reflect the views of a fringe minority (54.5%), whereas 33.7% of respondents believed that they reflect the broader public anger and concern over vaccine mandates during the COVID-19 pandemic (Fig. 2F) (Table S2).

### Multivariable regression analyses

A multivariable logistic regression analysis was performed to examine the relationship between sociodemographic characteristics and public approval of proof of COVID-19 vaccination mandates in Canada (Table S3). The odds ratios produced by the multivariate logistic regression revealed that none of the independent variables were statistically significant at a *α* = 0.05.

## Discussion

This study uniquely describes trends in attitudes towards vaccine certificates and mandates overtime in Canada, the site of some of the highest profile anti-vaccine certificate protests in the world. It is important to note that the attitudes examined of the participants are extensive in nature as they encompass an affective, behavioural, and cognitive (ABC) component. The ABC model of attitude has been shown to have importance during the COVID-19 pandemic [13]. For example, an individual who has been negatively impacted by the Freedom Convoy has had their attitude affected in all 3 domains. The question “Which of the following best describes your outlook on the COVID-19 outbreak?” invokes an affective component of attitude. While the question “Have you received at least one dose of a COVID-19 vaccine” invokes a behavioural component of attitude. The following question “How long do you expect it will take until things to return to normal?” invokes the cognitive component of attitude (Fig. 3).

We observed that: public attitudes towards vaccine certificates generally improved over time, public attitudes towards vaccine certificates became less polarized over time, trust towards government agencies (at the federal, provincial, and municipal levels) generally improved over time and the majority of study participants disapproved of the Freedom Convoy protestors, although there was a non-trivial proportion of those that supported them and these views were fairly polarized. However, the demographics that participated in this study may have influenced the results of these trends.

In interpreting the results of this study, it is important to recognize that the pandemic itself was dynamic scientifically and politically. At the outset of the pandemic, consideration was given to the use of proof of immunity or immunization certificates by the Canadian government but was initially rejected by the Prime Minister and the Chief Public Health Officer [14]. The development of a highly effective vaccine led to a re-evaluation of the need for proof of immunization certificates, but a voluntary vaccination approach was chosen [15]. As the virus mutated to the Delta variant in April 2021 which was both more infectious and created significant strains on health systems, the federal government and eventually provincial governments adopted the vaccine certificate program [16]. However, as the vaccine further mutated to the Omicron variant in November 2021, which was more infectious but with less morbidity and mortality, and for which vaccines protection against infection was substantially reduced, the argument for vaccine certificates lessened [17]. This led to the vaccine certificate protests – most notably the Freedom Convoy that occurred in Ottawa on January 29th, 2022, followed by provincial governments removing the requirements [12, 18].

We found that public attitudes towards vaccine certificates somewhat mirrored these scientific and political changes. Strong support for COVID-19 vaccine certificates and proof of vaccination measures fell from 66.0 to 43.1% and those who strongly opposed rose from 15.9 to 20.6%. However, interestingly the public expressed increasing discomfort over the pace of re-opening and trust in the federal government increased over this time. Attitudes towards provincial governments were, in general, more negative than attitudes towards local and federal governments. Our observation that public attitudes towards vaccine certificates seemed to have improved over time due to increased trust towards government agencies at the federal, provincial, and municipal levels is also demonstrated in the existing literature. However, the demographics of the sample participants may have contributed to this trend. Two large surveys conducted in the United Kingdom that examined public acceptance of privacy-tracking COVID-19 policies, including the implementation of immunity passports, reported that people’s perceived trust in the government’s intention and ability to securely manage their health data was the most important predictor of COVID-19 policy acceptance and associated with more favourable attitudes towards tracking policies [19]. Another global survey examining COVID-19 vaccine acceptance from participants in 19 different countries and found that those reporting higher levels of trust in their government were more likely to accept the vaccine and respond positively to an employer-enforced employee vaccination mandate than those with lower levels of trust [20]. The inverse relationship was also found to be true in a survey completed by Boguslavsky et al., where concerns over data privacy and cybersecurity related to low confidence in government was cited as a major proponent for low vaccine uptake after the introduction of QR code-based vaccine certificates in Russia [21].

Our findings reinforce the understanding that vaccine certificates and mandates were highly polarizing. They point to the importance of communication strategies to create trust that can influence the public’s reception of government-proposed public health initiatives and may help to facilitate implementation of vaccine certificates. They identify the challenges governments face of balancing supporting and understanding strongly held minority opinions, the views of the majority and the interests of public health. They also point to the dynamic nature of the pandemic and how this impacts policy decisions and public attitudes. In a global context, the outcomes hold implications that reach beyond Canada alone. The importance of trust in governmental and health institutions becomes pivotal during a pandemic. It was evident that a vocal minority had a considerable impact on the broader population. Additionally, it is important to recognize the impact of vaccine certificates on vaccination rates beyond Canada. The global literature reveals that vaccine certificates are linked to increased vaccine uptake [22]. The driving factors influencing the impact of vaccine certificates was trust in the safety, efficacy, and scientific basis of COVID-19 vaccines [22]. Our findings in combination with the global literature suggest the importance of policymakers across different nations developing effective communication strategies related to vaccine mandates. Shifts in public opinion surrounding vaccines and vaccine mandates can have a direct effect on future pandemic responses and vaccine mandate policies. These shifts should also be marked by shifts in public health messaging to the specific population of interest (often a vocal minority). The shift in messaging should cater to the targeted demographic group while simultaneously ensuring that individuals’ health freedoms are respected. Moving forward, it is important for governments to learn from the implementation of vaccine certificates given the potential for having to consider such policies for future pandemics. Data has demonstrated that this strategy did improve vaccination rates and reduced morbidity and mortality while improving health outcomes [3, 4, 6]. At the same time, these strategies challenged the social cohesion of societies. Developing strategies in the inter-pandemic period for how to use this intervention moving most effectively forward should be considered.

## Study Strengths & Limitations

There were a few limitations in this study. First, the study had relatively low response rates (ranging between 10.2–12.6% across the survey waves) and we did not examine differences in sociodemographic characteristics between responders and non-responders, which introduces the risk of non-response bias. The W28 (Emergency Act) survey, for example, coincided directly to when the Freedom Convoy in Ottawa occurred. The results of the W28 (Emergency Act) survey would evidently be impacted by such events. Individuals who are most impacted are most likely to voice their concerns and therefore be more willing to participate in such a survey. In addition, answers to questions posed on the survey will be highly polarized especially if one is negatively impacted by the events of the Freedom Convoy. Our demographic profile on individuals who did agree to partake in the study confirm this – the vast majority of individuals who did agree to partake in the study were individuals who did attain a higher education and are less likely to partake in the Freedom Convoy protest. Overall, many of the individuals who did agree to participate in the study had pursued a higher education. This may have skewed the data presented as it does not capture the general population.

Second, our surveys did not include questions pertaining to many important sociodemographic characteristics such as racial/ethnic groups, underlying medical conditions, prior vaccination history, and more, which may confound our observations on public attitudes towards vaccine mandates during the COVID-19 pandemic. Third, we did not assess for the risk of selection bias due to sociodemographic characteristics between the samples in each survey wave, which may further confound the observed associations. Fourth, the limited timeframe of our study between November 2021 to March 2022 precluded our ability to observe longer-term trends in public attitudes towards vaccine mandates, particularly during the earlier stages of the COVID-19 pandemic when such public health measures were more nascent, and the public may not yet have been acclimated as much to them. Fifth, there are concerns related to the external validity. Our sample was limited to English- or French-speaking adults living in Canada, thereby limiting the generalizability of our findings to other countries and non-English non-French-speaking adults. Also, we did not add a new cohort of participants throughout the study period which may have limited the diversity of opinions we have collected which may hinder external validity.

A strength in this study is that the surveys that were administered examined a broad cross-section of the Canadian population. This approach ensured in capturing diverse attitudes and perspectives. Another strength is that they surveys administered covered all regions of Canada, ensuring that perspectives that varied by Provinces and Territories were considered. Another strength of this study is that its longitudinal in nature. Surveys were administered over a period of time (November 2021 to March 2022) which allowed us to examine how attitudes towards COVID-19 vaccine mandates changed while encompassing changes in the political and social climate of the region.

## Conclusion

The current study offers valuable insights into the temporal evolution of public attitudes towards COVID-19 vaccine mandates introduced in Canada, as well as towards significant social and cultural events related to these vaccine mandates, such as the Freedom Convoy protest. These findings may guide the implementation of vaccine mandates in a more feasible and acceptable manner during future pandemics. Future studies involving longer-term follow-up and a more detailed investigation into the key themes identified (e.g., polarized views on vaccine mandates, trust towards government institutions, etc.) are warranted.

### Supplementary Information

**Supplementary Material 1.**

## Data Availability

No datasets were generated or analysed during the current study.

## References

[1] Ayouni I, Maatoug J, Dhouib W, Zammit N, Fredh SB, Ghammam R, Ghannem H (2021). Effective public health measures to mitigate the spread of COVID-19: a systematic review. BMC Public Health.

[2] Talic S, Shah S, Wild H, Gasevic D, Maharaj A, Ademi Z, Li X, Xu W, Mesa-Eguiagaray I, Rostron J (2021). Effectiveness of public health measures in reducing the incidence of covid-19, SARS-CoV-2 transmission, and covid-19 mortality: systematic review and meta-analysis. BMJ..

[3] Zhu DT, Mithani SS, Smith D, Wilson K (2023). The global impact of vaccine certificates on COVID-19 vaccine uptake: a scoping review.

[4] Mello MM, Opel JD, Benjamin RM, Callaghan T, DiResta R, Elharake JA, Flowers LC, Galvani AP, Salmon DA, Schwartz JL (2022). Effectiveness of vaccination mandates in improving uptake of COVID-19 vaccines in the USA. Lancet.

[5] Yasmin F, Najeeb J, Moeed A, Maeem U, Asghar MS, Chughtai NU, Yousaf Z, Seboka BT, Ullah I, Lin CY (2021). COVID-19 vaccine hesitancy in the United States: a systematic review. Front Public Health.

[6] Maquiling A, Jeevakanthan A, Fane BHM (2023). The effect of vaccine mandate announcements on vaccine uptake in Canada: an interrupted time series analysis. Vaccine..

[7] Hall MA, Studdert DM (2021). “Vaccine passport” certification — policy and ethical considerations. N Engl J Med.

[8] Sharun K, Tiwari R, Dhama K, Rabaan AA, Alhumaid S (2021). COVID-19 vaccination passport: prospects, scientific feasibility, and ethical concerns. Hum Vaccin Immunother.

[9] Gryzbowski A, Patryn RK, Sak J, Zagaja A (2017). Vaccination refusal. Autonomy and permitted coercion. Pathog Glob Health.

[10] Siegler AJ, Luisi N, Hall EW, Bradley H, Sanchez T, Lopman BA, Sullivan PS (2021). Trajectory of COVID-19 vaccine hesitancy over time and association of initial vaccine hesitancy with subsequent vaccination. JAMA..

[11] Fridman A, Gershon R, Gneezy A (2021). COVID-19 and vaccine hesitancy: a longitudinal study. PLoS One.

[12] Weeks C, Gray J. Ontario lifting COVID-19 mask mandates in most public settings, including schools, on march 21 - the globe and mail. The Globe and Mail Published 2022. Accessed October 5, 2023. https://www.theglobeandmail.com/canada/article-ontario-lifting-covid-19-mask-mandates-in-most-public-settings/.

[13] Chambon M, Dalege J, Elberse JE, van Harreveld F (2022). A psychological network approach to attitudes and preventive behaviors during pandemics: a COVID-19 study in the United Kingdom and the Netherlands. Soc Psychol Personal Sci.

[14] Baylis F, Kofler N. Why Canadians should fight tooth and nail against proof-of-immunity cards | CBC News. Canadian Broadcasting Corporation. https://www.cbc.ca/news/opinion/opinion-pandemic-coronavirus-immunity-passport-1.5551528. Published 2020. Accessed October 5, 2023.

[15] Immunize Canada. Is immunization mandatory in Canada? Immunize Canada. Published 2019. Accessed October 5, 2023. https://immunize.ca/immunization-mandatory-canada.

[16] Walkinshaw E (2011). Mandatory vaccinations: the Canadian picture. CMAJ..

[17] Schafer A. Lots of opposition but “a striking absence of good arguments” against vaccine passports, says ethicist | CBC news. Canadian Broadcasting Corporation Published 2021. Accessed October 5, 2023. https://www.cbc.ca/news/canada/manitoba/opinion-national-covid-19-vaccine-passports-arthur-schafer-1.6122386.

[18] Tasker JP. Thousands opposed to COVID-19 rules converge on Parliament Hill. Canadian Broadcasting Corporation. Published 2022. Accessed October 5, 2023. https://www.cbc.ca/news/politics/truck-convoy-protest-some-key-players-1.6332312.

[19] Lewandowsky S, Dennis S, Perfors A, Kashima Y, White JP, Garrett P (2021). Public acceptance of privacy-encroaching policies to address the COVID-19 pandemic in the United Kingdom. PLoS One.

[20] Lazarus JV, Ratzan SC, Palayew A, Gostin LO, Larson HJ, Rabin K, et al. A global survey of potential acceptance of a COVID-19 vaccine [published online ahead of print, 2020 Oct 20]. Nat Med. 2020:1–4. 10.1038/s41591-020-1124-9. 10.1038/s41591-020-1124-9 PMC7573523 33082575

[21] Boguslavsky DV, Sharova NP, Sharov KS (2022). Public policy measures to increase antiSARS-CoV-2 vaccination rate in Russia. Int J Environ Res Public Health.

[22] Zhu DT, Serhan M, Mithani SS, Smith D, Ang J, Thomas M, Wilson K (2023). The barriers, facilitators and association of vaccine certificates on COVID-19 vaccine uptake: a scoping review. Glob Health.

