# Supplementary material for: Public attitudes towards COVID-19 vaccine mandates and vaccine certificates in Canada: a time series study
Source: Arch Public Health. 2024 Mar 11;82:32. doi: 10.1186/s13690-024-01259-8 (PMC10926625; doi:10.1186/s13690-024-01259-8)
Supplement: Supplementary file 1 — Supplementary Material 1. [file 13690_2024_1259_MOESM1_ESM.docx]

**Appendix:**

**Table S1. Frequency data for recurring survey questions**

| Survey Responses | W25  n (%) | W26  n (%) | W27  n (%) | W28  n (%) | W29  n (%) | W30  n (%) | W31  n (%) | W32  n (%) |
| --- | --- | --- | --- | --- | --- | --- | --- | --- |
| **Total respondents (N)** | 1251 (100) | 1015 (100) | 1225 (100) | 1004 (100) | 1003 (100) | 1097 (100) | 1035 (100) | 1048 (100) |
| ***Q1: Which of the following best describes your outlook on the COVID-19 outbreak?*** | | | | | | | | |
| The worst is behind us | 871 (69.6) | 424 (41.8) | 661 (54.0) | 688 (68.5) | 696 (69.4) | 800 (72.9) | 374 (36.1) | 716 (68.3) |
| The worst is yet to come | 160 (12.8) | 320 (31.5) | 229 (18.7) | 146 (14.6) | 149 (14.9) | 129 (11.8) | 51 (4.9) | 132 (12.6) |
| Don’t know/no response | 220 (17.6) | 271 (26.7) | 335 (27.4) | 170 (16.9) | 158 (15.8) | 168 (15.3) | 610 (58.9) | 200 (19.1) |
| ***Q2: How long do you expect it will take until things to return to normal?*** | | | | | | | | |
| A month or less | 14 (1.1) | 9 (0.9) | 25 (2.0) | 37 (3.7) | 40 (4.0) | ~~–~~ | ~~–~~ | ~~–~~ |
| 1-3 months | 26 (2.1) | 11 (1.1) | 37 (3.0) | 54 (5.4) | 60 (6.0) | ~~–~~ | ~~–~~ | ~~–~~ |
| 3-6 months | 65 (5.2) | 46 (4.5) | 86 (7.0) | 87 (8.7) | 84 (8.4) | ~~–~~ | ~~–~~ | ~~–~~ |
| 6 months to a year | 228 (18.2) | 130 (12.8) | 195 (15.9) | 188 (18.7) | 162 (16.2) | ~~–~~ | ~~–~~ | ~~–~~ |
| 1-2 years | 384 (30.7) | 289 (28.5) | 317 (25.9) | 254 (25.3) | 232 (23.1) | ~~–~~ | ~~–~~ | ~~–~~ |
| 2 years or more | 278 (22.2) | 301 (29.7) | 286 (23.3) | 195 (19.4) | 169 (16.8) | ~~–~~ | ~~–~~ | ~~–~~ |
| Never | 191 (15.3) | 175 (17.2) | 183 (14.9) | 147 (14.6) | 201 (20.0) | ~~–~~ | ~~–~~ | ~~–~~ |
| Don’t know/no response | 65 (5.2) | 54 (5.3) | 96 (7.9) | 42 (4.2) | 55 (5.5) |  |  |  |
| ***Q3: How much stress would you say you are experiencing as a result of the COVID-19 pandemic?*** | | | | | | | | |
| None | 94 (7.5) | 61 (6.0) | 77 (6.3) | 73 (7.3) | 66 (6.6) | 111 (10.1) | 55 (5.3) | 61 (5.8) |
| Less than a moderate amount | 293 (23.4) | 200 (19.7) | 207 (16.9) | 180 (17.9) | 203 (20.2) | 273 (24.9) | 134 (12.9) | 136 (13.0) |
| A moderate amount | 454 (36.3) | 385 (37.9) | 438 (35.8) | 338 (33.7) | 352 (35.1) | 361 (32.9) | 177 (17.1) | 192 (18.3) |
| More than a moderate amount | 237 (18.9) | 186 (18.3) | 249 (20.3) | 191 (19.0) | 185 (18.4) | 187 (17.0) | 76 (7.3) | 68 (6.5) |
| A great deal | 168 (13.4) | 178 (17.5) | 250 (20.4) | 216 (21.5) | 191 (19.0) | 162 (14.8) | 77 (7.4) | 62 (5.9) |
| Don’t know/no response | 5 (0.4) | 5 (0.5) | 4 (0.3) | 6 (0.6) | 6 (0.6) | 3 (0.3) | 516 (49.9) | 529 (50.5) |
| ***Q4: Please rate the extent to which you agree or disagree with the following statement: “All Canadians should be required to wear masks in stores and while using public transit.”*** | | | | | | | | |
| Strongly disagree | 145 (11.6) | 142 (14.0) | 195 (15.9) | 195 (19.4) | 205 (20.4) | 225 (20.5) | 235 (22.7) | 203 (19.4) |
| Disagree | 51 (4.1) | 40 (3.9) | 54 (4.4) | 64 (6.4) | 52 (5.2) | 74 (6.7) | 67 (6.5) | 99 (9.4) |
| Neither agree nor disagree | 83 (6.6) | 61 (6.0) | 72 (5.9) | 89 (8.9) | 92 (9.2) | 127 (11.6) | 85 (8.2) | 135 (12.9) |
| Agree | 146 (11.7) | 114 (11.2) | 137 (11.2) | 127 (12.6) | 157 (15.7) | 204 (18.6) | 228 (22.0) | 200 (19.1) |
| Strongly agree | 822 (65.7) | 651 (64.1) | 747 (61.0) | 522 (52.0) | 489 (48.8) | 460 (41.9) | 409 (39.5) | 405 (38.6) |
| Don’t know/no response | 4 (0.3) | 7 (0.7) | 20 (1.7) | 7 (0.7) | 8 (0.8) | 7 (0.6) | 11 (1.1) | 6 (0.6) |
| ***Q5: Have you received at least one dose of a COVID-19 vaccine?*** | | | | | | | | |
| Yes, one dose | 9 (0.7) | 13 (1.3) | 15 (1.2) | 8 (0.8) | 5 (0.5) | 7 (0.6) | 10 (1.0) | 10 (1.0) |
| Yes, two or more doses | 1122 (89.7) | 890 (87.7) | 347 (28.3) | 196 (19.5) | 189 (18.8) | 176 (16.0) | 167 (16.1) | 146 (13.9) |
| Yes, three or more doses | 84 (6.7) | 191 (18.8) | 728 (59.4) | 705 (70.2) | 705 (70.3) | 809 (73.7) | 756 (73.0) | 807 (77.0) |
| No | 90 (7.2) | 85 (8.4) | 108 (8.8) | 74 (7.4) | 72 (7.2) | 80 (7.3) | 82 (7.9) | 65 (6.2) |
| Don’t know/no response | 30 (2.4) | 27 (2.7) | 27 (2.2) | 21 (2.1) | 32 (3.2) | 25 (2.3) | 20 (1.9) | 20 (1.9) |
| ***Q6: Would you be willing to receive a third dose of a COVID-19 vaccine?*** | | | | | | | | |
| Yes | 874 (69.9) | 590 (58.1) | 219 (17.9) | 77 (7.7) | 77 (7.7) | 66 (6.0) | 51 (4.9) | 45 (4.3) |
| No | 94 (7.5) | 62 (6.1) | 86 (7.0) | 88 (8.8) | 91 (9.1) | 80 (7.3) | 88 (8.5) | 76 (7.3) |
| I already have a third dose | 84 (6.7) | 191 (18.8) | 728 (59.4) | 705 (70.2) | 705 (70.3) | 809 (73.7) | 756 (73.0) | 807 (77.0) |
| Don’t know/no response | 199 (15.9) | 172 (16.9) | 192 (15.7) | 134 (13.4) | 130 (13.0) | 142 (13.0) | 140 (13.6) | 120 (11.5) |
| ***Q7: To what extent do you support or oppose requiring Canadians to carry proof of vaccination in order to attend public events like concerts and sporting events?*** | | | | | | | | |
| Strongly oppose | 199 (15.9) | 175 (17.2) | 78 (6.4) | 234 (23.3) | 238 (23.7) | 241 (22.0) | 251 (24.3) | 216 (20.6) |
| Oppose | 40 (3.2) | 34 (3.3) | 30 (2.4) | 45 (4.5) | 47 (4.7) | 52 (4.7) | 63 (6.1) | 70 (6.7) |
| Neither support nor oppose | 70 (5.6) | 44 (4.3) | 26 (2.1) | 48 (4.8) | 88 (8.8) | 109 (9.9) | 91 (8.8) | 103 (9.8) |
| Support | 112 (9.0) | 84 (8.3) | 39 (3.2) | 111 (11.1) | 149 (14.9) | 160 (14.6) | 180 (17.4) | 200 (19.1) |
| Strongly support | 826 (66.0) | 674 (66.4) | 231 (18.9) | 563 (56.1) | 475 (47.4) | 531 (48.4) | 442 (42.7) | 452 (43.1) |
| Don’t know/no response | 4 (0.3) | 4 (0.4) | 821 (67.0) | 3 (0.3) | 6 (0.6) | 4 (0.4) | 8 (0.8) | 7 (0.7) |
| ***Q8: All things considered, do you believe your province is re-opening too quickly, too slowly, or at about the right pace?*** | | | | | | | | |
| Too slowly | 175 (14.0) | — | — | 258 (25.7) | 215 (21.4) | 216 (19.7) | 199 (19.2) | 155 (14.8) |
| Slower than desired | 66 (5.3) | — | — | 110 (11.0) | 73 (7.3) | 100 (9.1) | 63 (6.1) | 74 (7.1) |
| About the right pace | 627 (50.1) | — | — | 355 (35.4) | 331 (33.0) | 379 (34.5) | 346 (33.4) | 351 (33.5) |
| Quicker than desired | 210 (16.8) | — | — | 124 (12.4) | 149 (14.9) | 186 (17.0) | 210 (20.3) | 223 (21.3) |
| Too quickly | 155 (12.4) | — | — | 136 (13.5) | 208 (20.7) | 190 (17.3) | 196 (18.9) | 230 (21.9) |
| Don’t know/no response | 18 (1.4) | — | — | 21 (2.1) | 27 (2.7) | 26 (2.4) | 21 (2.0) | 15 (1.4) |
| ***Q9: To what extent do you trust the government of Canada as a whole?*** | | | | | | | | |
| Strongly disapprove | — | — | 265 (21.6) | 266 (26.5) | 260 (25.9) | 252 (23.0) | 224 (21.6) | 188 (17.9) |
| Disapprove | — | — | 189 (15.4) | 149 (14.8) | 117 (11.7) | 141 (12.9) | 124 (12.0) | 148 (14.1) |
| Neither approve nor disapprove | — | — | 226 (18.4) | 130 (12.9) | 144 (14.4) | 165 (15.0) | 134 (12.9) | 158 (15.1) |
| Approve | — | — | 388 (31.7) | 303 (30.2) | 305 (30.4) | 325 (29.6) | 354 (34.2) | 389 (37.1) |
| Strongly approve | — | — | 143 (11.7) | 150 (14.9) | 170 (16.9) | 206 (18.8) | 190 (18.4) | 159 (15.2) |
| Don’t know/no response | — | — | 14 (1.2) | 6 (0.6) | 7 (0.7) | 8 (0.7) | 9 (0.9) | 6 (0.6) |
| ***Q10: To what extent do you trust the Public Health Agency of Canada?*** | | | | | | | | |
| Strongly disapprove | — | — | 201 (16.4) | 186 (18.5) | 176 (17.5) | 173 (15.8) | 165 (15.9) | 136 (13.0) |
| Disapprove | — | — | 142 (11.6) | 132 (13.1) | 100 (10.0) | 113 (10.3) | 119 (11.5) | 126 (12.0) |
| Neither approve nor disapprove | — | — | 207 (16.9) | 169 (16.8) | 167 (16.7) | 186 (17.0) | 147 (14.2) | 167 (15.9) |
| Approve | — | — | 440 (35.9) | 309 (30.8) | 327 (32.6) | 361 (32.9) | 336 (32.5) | 379 (36.2) |
| Strongly approve | — | — | 215 (17.6) | 184 (18.3) | 213 (21.2) | 244 (22.2) | 250 (24.2) | 225 (21.5) |
| Don’t know/no response | — | — | 20 (1.7) | 24 (2.4) | 20 (2.0) | 20 (1.8) | 18 (1.7) | 15 (1.4) |
| ***Q11: To what extent do you trust your provincial government?*** | | | | | | | | |
| Strongly disapprove | — | — | 350 (28.6) | 283 (28.2) | 306 (30.5) | 298 (27.2) | 297 (28.7) | 293 (28.0) |
| Disapprove | — | — | 264 (21.6) | 204 (20.3) | 188 (18.7) | 200 (18.2) | 203 (19.6) | 195 (18.6) |
| Neither approve nor disapprove | — | — | 163 (13.3) | 147 (14.6) | 145 (14.5) | 152 (13.9) | 118 (11.4) | 117 (11.2) |
| Approve | — | — | 290 (23.7) | 249 (24.8) | 212 (21.1) | 259 (23.6) | 243 (23.5) | 286 (27.3) |
| Strongly approve | — | — | 148 (12.1) | 115 (11.5) | 145 (14.5) | 182 (16.6) | 166 (16.0) | 155 (14.8) |
| Don’t know/no response | — | — | 10 (0.8) | 6 (0.6) | 7 (0.7) | 6 (0.5) | 8 (0.8) | 2 (0.2) |
| ***Q12: To what extent do you trust your municipal government?*** | | | | | | | | |
| Strongly disapprove | — | — | 141 (11.5) | 136 (13.5) | 127 (12.7) | 127 (11.6) | 114 (11.0) | 111 (10.6) |
| Disapprove | — | — | 133 (10.9) | 110 (11.0) | 113 (11.3) | 120 (10.9) | 123 (11.9) | 120 (11.5) |
| Neither approve nor disapprove | — | — | 372 (30.4) | 267 (26.6) | 302 (30.1) | 319 (29.1) | 266 (25.7) | 2654 (25.3) |
| Approve | — | — | 339 (27.7) | 285 (28.4) | 244 (24.3) | 297 (27.1) | 308 (29.8) | 333 (31.8) |
| Strongly approve | — | — | 150 (12.2) | 137 (13.6) | 146 (14.6) | 159 (14.5) | 169 (16.3) | 167 (15.9) |
| Don’t know/no response | — | — | 90 (7.4) | 69 (6.9) | 71 (7.1) | 75 (6.8) | 55 (5.3) | 52 (5.0) |
| ***Q13: Some people say that pandemic-related restrictions have gone on long enough, it is time to bring an end to COVID-19 restrictions. Others say that there are still reasons to be careful and we should keep some restrictions in place for the time being. Which of the following statements comes closest to your own point of view?*** | | | | | | | | |
| It is time to bring an end to COVID-19 related restrictions | — | — | — | 400 (39.8) | 408 (40.7) | 445 (40.6) | 429 (41.4) | 431 (41.1) |
| We should keep COVID-19 related restrictions in place for the time being | — | — | — | 560 (55.8) | 551 (54.9) | 591 (53.9) | 541 (52.3) | 549 (52.4) |
| Don’t know/no response | — | — | — | 44 (4.4) | 44 (4.4) | 61 (5.6) | 65 (6.3) | 68 (6.5) |

**Table S2. Frequency data for Freedom Convoy questions during W28**

| Survey Responses | n (%) |
| --- | --- |
| **Total respondents (N)** | 1004 (100) |
| ***Q1: On January 15, the federal government ended exemptions to vaccine mandates for truck drivers, meaning that truckers entering Canada from the United States will need to either show proof of vaccination against COVID-19 or quarantine for two weeks upon arrival. The United States introduced similar requirements for Canadian truckers on January 22. To what extent do you support or oppose this measure?*** | |
| Strongly oppose | 142 (14.1) |
| Oppose | 29 (2.9) |
| Neither support nor oppose | 38 (3.8) |
| Support | 46 (4.6) |
| Strongly support | 246 (24.5) |
| Don’t know/no response | 503 (50.1) |
| ***Q2: As you may know, a "Freedom Convoy" of truckers and other demonstrators has travelled to Ottawa. The protest was sparked by the vaccine mandate for cross-border truckers, but it has since grown to a push for an end to all vaccine mandates nationwide. How closely have you been following this protest?*** | |
| Very closely | 485 (48.3) |
| Somewhat closely | 443 (44.1) |
| Not at all | 72 (7.2) |
| Don’t know/no response | 4 (0.4) |
| ***Q3: Based on what you know, to what extent do you support or oppose this Freedom Convoy protest?*** | |
| Strongly oppose | 521 (51.9) |
| Oppose | 90 (9.0) |
| Neither support nor oppose | 46 (4.6) |
| Support | 66 (6.6) |
| Strongly support | 204 (20.3) |
| Don’t know/no response | 77 (7.7) |
| ***Q4: I would support a similar Freedom Convoy protest in my community*** | |
| Strongly disagree | 589 (58.7) |
| Disagree | 49 (4.9) |
| Neither agree nor disagree | 54 (5.4) |
| Agree | 56 (5.6) |
| Strongly agree | 176 (17.5) |
| Don’t know/no response | 80 (8.0) |
| ***Q5: I identify with the Freedom Convoy convoy protestors*** | |
| Strongly disagree | 579 (57.7) |
| Disagree | 52 (5.2) |
| Neither agree nor disagree | 79 (7.9) |
| Agree | 69 (6.9) |
| Strongly agree | 144 (14.3) |
| Don’t know/no response | 81 (8.1) |
| ***Q6: I really worry that these sorts of Freedom Convoy protests are reflective of authoritarian populism, similar to what has been going on in the United States*** | |
| Strongly disagree | 209 (20.8) |
| Disagree | 42 (4.2) |
| Neither agree nor disagree | 68 (6.8) |
| Agree | 136 (13.5) |
| Strongly agree | 446 (44.4) |
| Don’t know/no response | 103 (10.3) |
| ***Q7: Some of the Freedom Convoy organizers have created a "memorandum of understanding" calling on the Governor General to either override vaccine mandates across the country or resign. To what extent would you support or oppose the Governor General intervening on the matter of vaccine mandates?*** | |
| Strongly oppose | 610 (60.8) |
| Oppose | 53 (5.3) |
| Neither support nor oppose | 90 (9.0) |
| Support | 59 (5.9) |
| Strongly support | 143 (14.2) |
| Don’t know/no response | 49 (4.9) |
| ***Q8: Some people say the convoy protest is a genuine reflection of broad public anger and concerns, while others say it reflects the views of a fringe minority. Which of these statements comes closest to your own point of view?*** | |
| The protest reflect public anger and concern | 338 (33.7) |
| The protest reflects the views of a fringe minority | 547 (54.5) |
| Don’t know/no response | 119 (11.9) |
| ***Q9: With regards to the convoy protest, which of these statements comes closest to your own point of view with regards to authority?*** | |
| Questioning authority | 385 (38.3) |
| Neither | 355 (35.4) |
| Obedience | 194 (19.3) |
| Don’t know/no response | 70 (7.0) |
| ***Q10: With regards to the convoy protest, which of these statements comes closest to your own point of view with regards to rationality?*** | |
| Morality | 222 (22.1) |
| Neither | 126 (12.5) |
| Reason and evidence | 576 (57.4) |
| Don’t know/no response | 80 (8.0) |
| ***Q11: With regards to the convoy protest, which of these statements comes closest to your own point of view with regards to expression?*** | |
| Creativity | 377 (37.5) |
| Neither | 219 (21.8) |
| Good behaviour | 329 (32.8) |
| Don’t know/no response | 79 (7.9) |
| ***Q12: With regards to the convoy protest, which of these statements comes closest to your own point of view with regards to order?*** | |
| Order | 173 (17.2) |
| Neither | 187 (18.6) |
| Openness | 570 (56.8) |
| Don’t know/no response | 74 (7.4) |
| ***Q13: With regards to the convoy protest, which of these statements comes closest to your own point of view with regards to trust?*** | |
| Trust in science and experts | 642 (63.9) |
| Neither | 190 (18.9) |
| Skepticism of science and experts | 125 (12.5) |
| Don’t know/no response | 47 (4.7) |

**Table S3. Multivariate Logistic Regression of Sociodemographic Characteristics Predicting Support for Proof of Vaccination (Waves 25–32).**

| Demographic factor | Week 25 | | Week 26 | | Week 29 | | Week 30 | | Week 31 | | Week 32 | |
| --- | --- | --- | --- | --- | --- | --- | --- | --- | --- | --- | --- | --- |
|  | B | OR (95% CI) | B | OR  (95% CI) | B | OR  (95% CI) | B | OR  (95% CI) | B | OR  (95% CI) | B | OR  (95% CI) |
| Age (years) |  |  |  |  |  |  |  |  |  |  |  |  |
| 18–24 (ref) | 0.000 (ref) | 1.00 (ref) | 0.000 (ref) | 1.00 (ref) | 0.000 (ref) | 1.00 (ref) | 0.000 (ref) | 1.00 (ref) | 0.000 (ref) | 1.00 (ref) | 0.000 (ref) | 1.00 (ref) |
| 25–34 | -0.122 | 0.885  (0.296, 2.651) | -0.854 | 0.426  (0.124, 1.458) | -0.172 | 0.842  (0.342, 2.072) | 0.161 | 1.174 (0.424, 3.253) | 0.882 | 2.416 (0.585, 9.981) | -0.524 | 0.592  (0.203, 1.727) |
| 35–44 | -0.395 | 0.674  (0.296, 2.056) | -0.980 | 0.375  (0.108, 1.308) | -0.088 | 0.916  (0.369, 2.273) | 0.085 | 1.089  (0.393, 3.022) | 0.983 | 2.674  (0.632, 11.304) | -0.556 | 0.574  (0.196, 1.680) |
| 45–54 | -0.215 | 0.806  (0.263, 2.470) | -0.947 | 0.388  (0.112, 1.340) | -0.212 | 0.809  (0.323, 2.027) | 0.015 | 1.015 (0.368, 2.797) | 1.085 | 2.958 (0.706, 12.391) | -1.121 | 0.326  (0.110, 0.971) |
| 55–64 | -0.920 | 0.399  (0.130, 1.220) | -1.805 | 0.165  (0.047, 0.579) | -0.838 | 0.433  (0.170, 1.100) | -0.591 | 0.554  (0.198, 1.545) | 0.767 | 2.154  (0.513, 9.035) | -1.097 | 0.334  (0.111, 1.000) |
| 65+ | -1.898 | 0.150  (0.047, 0.479) | -2.606 | 0.074  (0.020, 0.269) | -1.501 | 0.223  (0.086, 0.576) | -1.413 | 0.243  (0.086, 0.687) | -0.384 | 0.681  (0.158, 2.937) | -1.779 | 0.169  (0.057, 0.501) |
| Don’t know/  No response | -0.690 | 0.502  (0.078, 3.212) | -0.528 | 0.590  (0.148, 2.349) | -0.311 | 0.732  (0.230, 2.328) | 0.338 | 1.402  (0.414, 4.748) | 1.252 | 3.496  (0.699, 17.479) | -0.897 | 0.408  (0.103, 1.612) |
| Gender |  |  |  |  |  |  |  |  |  |  |  |  |
| Male (ref) | 0.000 (ref) | 1.00 (ref) | 0.000 (ref) | 1.00 (ref) | 0.000 (ref) | 1.00 (ref) | 0.000 (ref) | 1.00 (ref) | 0.000 (ref) | 1.00 (ref) | 0.000 (ref) | 1.00 (ref) |
| Female | -0.699 | 0.497  (0.355, 0.697) | -0.196 | 0.822  (0.570, 1.187) | -0.502 | 0.605  (0.437, 0.839) | -0.368 | 0.692  (0.499, 0.959) | -0.423 | 0.655  (0.480, 0.895) | -0.749 | 0.473  (0.344, 0.650) |
| Other | -0.474 | 0.622  (0.081, 4.801) | 0.564 | 1.758  (0.463, 6.671) | 0.909 | 2.482  (0.571, 10.792) | -0.439 | 0.645  (0.144, 2.879) | -1.095 | 0.334  (0.067, 1.680) | -2.144 | 0.117  (0.014, 1.013) |
| Don’t know/  No response | 0.988 | 0.502  (0.078, 3.212) | 0.637 | 1.891  (0.368, 9.712) | 0.614 | 1.847  (0.438, 7.784) | -0.397 | 0.672 (0.160, 2.820) | 0.618 | 0.539  (0.044, 6.651) | 0.607 | 1.834  (0.279, 12.063) |
| Rurality |  |  |  |  |  |  |  |  |  |  |  |  |
| Urban (ref) | 0.000 (ref) | 1.00 (ref) | 0.000 (ref) | 1.00 (ref) | 0.000 (ref) | 1.00 (ref) | 0.000 (ref) | 1.00 (ref) | 0.000 (ref) | 1.00 (ref) | 0.000 (ref) | 1.00 (ref) |
| Suburban | 0.028 | 1.028  (0.695, 1.521) | 0.376 | 1.456  (0.961, 2.207) | 0.167 | 1.182  (0.813, 1.718) | 0.063 | 1.065 (0.739, 1.536) | 0.460 | 1.584  (1.113, 2.256) | 0.463 | 1.589  (1.110, 2.275) |
| Rural | 0.922 | 2.515  (1.658, 3.815) | 0.918 | 2.503  (1.566, 4.002) | 0.900 | 2.461  (1.606, 3.770) | 0.699 | 2.012  (1.322, 3.063) | 1.086 | 2.961  (1.968, 4.455) | 1.054 | 2.870  (1.889, 4.361) |
| Don’t know/  No response | 0.988 | 2.685  (0.492, 14.638) | 1.388 | 4.006  (0.479, 33.509) | 0.159 | 1.173 (0.266, 5.163) | 0.040 | 1.041 (0.314, 3.456) | 1.783 | 5.950 (1.497, 23.645) | 2.379 | 10.799 (0.942, 123.808) |
| Province |  |  |  |  |  |  |  |  |  |  |  |  |
| Ontario (ref) | 0.000 (ref) | 1.00 (ref) | 0.000 (ref) | 1.00 (ref) | 0.000 (ref) | 1.00 (ref) | 0.000 (ref) | 1.00 (ref) | 0.000 (ref) | 1.00 (ref) | 0.000 (ref) | 1.00 (ref) |
| Alberta | 0.607 | 1.834  (1.123, 2.997) | 0.341 | 1.406  (0.818, 2.416) | 0.073 | 1.076  (0.650, 1.781) | 0.226 | 1.254  (0.769, 2.045) | 0.273 | 1.314  (0.801, 2.155) | 0.627 | 1.872  (1.151, 3.043) |
| Saskatchewan | -0.537 | 0.585  (0.187, 1.827) | 0.277 | 1.319  (0.515, 3.374) | -0.274 | 0.760  (0.290, 1.996) | -0.262 | 0.770  (0.317, 1.872) | -0.091 | 0.913  (0.378, 2.205) | 0.127 | 1.135  (0.511, 2.519) |
| Manitoba | 0.237 | 1.268  (0.545, 2.951) | -0.413 | 0.662  (0.208, 2.109) | 0.358 | 1.431  (0.530, 3.862) | -0.200 | 0.819  (0.305, 2.198) | -0.581 | 0.559  (0.231, 1.358) | -0.554 | 0.575  (0.231, 1.431) |
| British  Columbia | 0.078 | 1.081  (0.659, 1.773) | 0.009 | 1.009  (0.576, 1.764) | -0.185 | 0.831  (0.508, 1.360) | -0.483 | 0.617  (0.373, 1.020) | 0.052 | 1.053  (0.671, 1.652) | 0.059 | 1.061  (0.653, 1.723) |
| Quebec | 0.001 | 1.001  (0.643, 1.557) | -0.108 | 0.898  (0.540, 1.492) | 0.042 | 1.043  (0.670, 1.623) | 0.026 | 1.026  (0.677, 1.556) | 0.014 | 1.015  (0.648, 1.587) | 0.392 | 1.480  (0.972, 2.252) |
| New  Brunswick | -1.239 | 0.290  (0.069, 1.224) | 0.535 | 1.708  (0.693, 4.211) | 0.210 | 1.234  (0.499, 3.050) | 0.677 | 1.968  (0.712, 5.436) | -0.329 | 0.720  (0.273, 1.895) | -1.078 | 0.340  (0.084, 1.378) |
| Nova Scotia | -0.239 | 0.787  (0.328, 1.891) | 0.222 | 1.248  (0.511, 3.049) | 0.005 | 1.005  (0.431, 2.344) | 0.337 | 1.400  (0.611, 3.212) | 0.198 | 1.219  (0.580, 2.563) | -0.188 | 0.829  (0.336, 2.045) |
| Prince Edward  Island | — | — | — | — | — | — | — | — | 0.394 | 1.484  (0.124, 17.730) | 0.749 | 2.115  (0.095, 47.341) |
| Newfoundland  and Labrador | -0.954 | 0.385  (0.081, 1.841) | 0.330 | 1.391  (0.247, 7.835) | 0.160 | 1.174  (0.246, 5.592) | — | — | -1.388 | 0.249  (0.051, 1.230) | -0.002 | 0.998  (0.241, 4.126) |
| Yukon | — | — | 0.555 | 1.742  (0.348, 8.724) | -0.655 | 0.519  (0.072, 3.736) | -0.498 | 0.608  (0.143, 2.579) | 0.965 | 2.624  (0.644, 10.700) | 1.500 | 4.480  (1.164, 17.239) |
| Northwest  Territories | — | — | 0.364 | 1.439  (0.172, 12.014) | 0.164 | 1.178  (0.112, 12.420) | 1.117 | 3.055  (0.390, 23.952) | 0.229 | 1.258  (0.306, 5.168) | — | — |
| Nunavut | — | — | — | — | 0.906 | 2.474  (0.128, 47.970) | 1.024 | 2.783  (0.145, 53.505) | -0.408 | 0.665  (0.048, 9.152) | — | — |
| Don’t know/  No response | — | — | — | — | 1.445 | 4.244  (0.260, 69.150) | — | — | -1.642 | 0.194  (0.006, 6.742) | — | — |
| Income |  |  |  |  |  |  |  |  |  |  |  |  |
| <$10,000  (ref) | 0.000 (ref) | 1.00 (ref) | 0.000 (ref) | 1.00 (ref) | 0.000 (ref) | 1.00 (ref) | 0.000 (ref) | 1.00 (ref) | 0.000 (ref) | 1.00 (ref) | 0.000 (ref) | 1.00 (ref) |
| <10,000–  $19,999 | -0.555 | 0.574  (0.094, 3.506) | 0.879 | 2.409  (0.196, 29.618) | -0.770 | 0.463  (0.089, 2.417) | 10.451 | 1.571  (0.122, 20.263) | -2.657 | 0.070  (0.006, 0.780) | -0.030 | 0.971  (0.112, 8.387) |
| <20,000–  $29,999 | -0.707 | 0.493  (0.103, 2.368) | 1.604 | 4.973  (0.424, 58.300) | 0.281 | 1.325  (0.317, 5.542) | 0.088 | 1.092  (0.086, 13.837) | -0.846 | 0.429  (0.073, 2.537) | -0.667 | 0.513  (0.074, 3.545) |
| <30,000–  $39,999 | -1.299 | 0.273  (0.052, 1.438) | -0.872 | 0.418  (0.021, 8.367) | -0.492 | 0.611  (0.152, 2.452) | 0.210 | 1.234  (0.103, 14.817) | -1.075 | 0.341  (0.059, 1.990) | -0.761 | 0.467  (0.069, 3.147) |
| <40,000–  $49,999 | -0.190 | 0.827  (0.195, 3.505) | 1.619 | 5.047  (0.488, 52.199) | 0.130 | 1.138  (0.290, 4.465) | 0.497 | 1.644  (0.143, 18.868) | -0.983 | 0.374  (0.066, 2.112) | -0.853 | 0.426  (0.063, 2.863) |
| <50,000–  $59,999 | -0.483 | 0.617  (0.146, 2.614) | 1.305 | 3.689  (0.364, 37.380) | 0.188 | 1.207  (0.316, 4.607) | 1.007 | 2.736  (0.241, 31.061) | -0.396 | 0.673  (0.128, 3.534) | -0.870 | 0.419  (0.066, 2.670) |
| <60,000–  $79,999 | -0.503 | 0.605  (0.151, 2.414) | 1.672 | 5.324  (0.552, 51.392) | 0.038 | 1.038  (0.289, 3.732) | 0.273 | 1.314  (0.119, 14.486) | -0.582 | 0.559  (0.110, 2.846) | -0.964 | 0.381  (0.063, 2.315) |
| <80,000–  $99,999 | -0.432 | 0.649  (0.164, 2.569) | 1.803 | 6.069  (0.639, 57.608) | -0.025 | 0.975  (0.268, 3.546) | 0.509 | 1.663  (0.151, 18.313) | -0.937 | 0.392  (0.076, 2.023) | -0.844 | 0.430  (0.071, 2.615) |
| <100,000–  $119,999 | 0.541 | 0.582  (0.147, 2.296) | 1.300 | 3.668  (0.377, 35.650) | 0.154 | 1.167  (0.326, 4.175) | 0.499 | 1.647  (0.151, 17.975) | -0.773 | 0.462  (0.090, 2.358) | -0.950 | 0.387  (0.064, 2.337) |
| <120,000–  $159,999 | -0.096 | 0.909  (0.231, 3.569) | 1.176 | 3.242  (0.337, 31.225) | 0.173 | 1.189  (0.329, 4.290) | 0.687 | 1.988  (0.181, 21.889) | -0.884 | 0.413  (0.082, 2.088) | -0.835 | 0.434  (0.073, 2.590) |
| <160,000–  $219,999 | -0.619 | 0.539  (0.131, 2.211) | 1.167 | 3.212  (0.325, 31.709) | 0.272 | 1.313 (0.358, 4.811) | 0.114 | 1.121  (0.101, 12.405) | -0.758 | 0.469  (0.091, 2.416) | -0.969 | 0.379  (0.061, 2.340) |
| 220,000+ | -0.548 | 0.578  (0.134, 2.486) | 0.997 | 2.711  (0.268, 27.425) | 0.243 | 1.275  (0.319, 5.098) | 0.234 | 1.263  (0.111, 14.436) | -0.858 | 0.424  (0.082, 2.201) | -0.791 | 0.454  (0.073, 2.817) |
| Don’t know/  No response | -0.161 | 0.851  (0.221, 3.280) | 1.149 | 3.156  (0.334, 29.805) | -0.185 | 0.831  (0.230, 3.002) | 0.359 | 1.432  (0.131, 15.696) | -1.044 | 0.352  (0.068, 1.810) | -0.498 | 0.608  (0.101, 3.656) |
| Minority |  |  |  |  |  |  |  |  |  |  |  |  |
| None (ref) | 0.000 (ref) | 1.00 (ref) | 0.000 (ref) | 1.00 (ref) | 0.000 (ref) | 1.00 (ref) | 0.000 (ref) | 1.00 (ref) | 0.000 (ref) | 1.00 (ref) | 0.000 (ref) | 1.00 (ref) |
| Indigenous | -0.179 | 0.836  (0.321, 2.181) | -0.295 | 0.745  (0.272, 2.038) | -0.025 | 0.976  (0.389, 2.447) | -0.345 | 0.708  (0.295, 1.703) | 0.554 | 1.740  (0.735, 4.119) | 0.317 | 1.373  (0.540, 3.491) |
| Disabled | -0.118 | 0.889  (0.455, 1.735) | -0.547 | 0.579  (0.276, 1.212) | -0.433 | 0.648  (0.341, 1.232) | -0.484 | 0.617  (0.331, 1.150) | -0.368 | 0.692  (0.393, 1.220) | -0.296 | 0.743  (0.412, 1.340) |
| 2SLGBTQ+ | -0.731 | 0.481  (0.220, 1.051) | -1.463 | 0.232  (0.084, 0.640) | -0.529 | 0.589  (0.266, 1.303) | -0.991 | 0.371  (0.148, 0.929) | -0.712 | 0.491  (0.229, 1.053) | -0.320 | 0.726  (0.348, 1.515) |
| Visible  minority | 0.322 | 1.380  (0.829, 2.297) | 0.138 | 1.148  (0.681, 1.934) | 0.284 | 1.328  (0.823, 2.144) | -0.008 | 0.992  (0.596, 1.652) | -0.065 | 0.937  (0.589, 1.493) | -0.158 | 0.854  (0.521, 1.401) |
| Don’t know/  No response | 1.388 | 4.007  (1.934, 8.299) | 1.228 | 3.416  (1.652, 7.062) | 1.646 | 5.184  (2.670, 10.066) | 1.769 | 5.864  (2.846, 12.082) | 1.538 | 4.658  (2.268, 9.563) | 1.437 | 4.207  (1.967, 8.996) |

Abbreviations: B is the regression coefficient, OR is the odds ratio, CI is the confidence interval.

Note: Weeks 27 and 28 were omitted from multivariate analyses due to the substantial amount of missing data.

Note: Horizontal dashes denote unavailable parameter estimates due to missing data.
